# Organization and regional distribution of centers for the management of children and adolescents with diabetes in Italy

**DOI:** 10.1186/s13052-015-0179-6

**Published:** 2015-10-08

**Authors:** Chiara Giorgetti, Lucia Ferrito, Federica Zallocco, Antonio Iannilli, Valentino Cherubini, Study Group for Diabetes of ISPED

**Affiliations:** S.O.D. Pediatric Diabetes, Department of Women’s and Children’s Health, Salesi Hospital, Azienda Ospedaliero-Universitaria, Ospedali Riuniti Ancona, Presidio “G. Salesi”, Via Corridoni 11, 60100 Ancona, Italy

**Keywords:** Type 1 diabetes, Prevalence, Regional legislation, Pediatric diabetes centers, Organization of care

## Abstract

**Background:**

The incidence of type 1 diabetes in childhood is increasing by 3 % per year, placing growing demands on healthcare professionals and medical expenditures. Aim of this study wars to assess the organization of care to children with diabetes in Italy.

**Methods:**

During 2012 a structured questionnaire was sent to all of the members of Italian Society of Paediatric Endocrinology and Diabetology (ISPED). Questions examined organizational structure of Centers, personnel dedicated to the care of children with diabetes, number of subjects followed, local legal legislation supporting centres.

**Results:**

A total of 68 centers taking care to 15,563 children and adolescents with diabetes under 18 years of age were identified with a prevalence of 1.4 per 1,000 people. A wide variation in the organizational background was also reported. Fourty-four centers were organized as outpatient departments, 17 as simple units, 5 as complex units and 2 as simple departmental structures. Most centers had a multidisciplinary team. Ten out of twenty Italian regions had introduced supportive regional legislation, but it was fully applied only in six of them.

**Conclusion:**

Great differences between regions were found in organizational structures, staffing levels and supportive legislation. The national legislation on diabetes was broadly implemented throughout the country regions. Further efforts are needed to improve standards and consistency of pediatric diabetes care in Italy.

## Introduction

Type 1 diabetes (T1D) is one of the most common endocrine and metabolic conditions in childhood, showing an increasing incidence rate of about 3 % per year during the last two decades. During the period 1990–2003 the incidence rate in Italy was 12.26 per 100,000 person-years, with an increasing temporal trend of 2.94 % per year [95 % CI 2.22–3.67] [1]. Maintaining this trend, a doubling of new cases of T1D in children is expected between 2005 and 2020, with increasing burden for the families and the health care system [2]. Moreover, a further burden for the health care system derives from the growing number of immigrant children living in Italy, as they usually have a younger age at diagnosis and a significantly poorer metabolic control compared with western patients [3]. In different studies micro and macrovascular complications have been reported with various frequencies, even a short time after the onset of diabetes, but mainly depending on diabetes duration and glycemic control [4–7]. Retinopathy, nephropathy and neuropathy have rarely been reported in prepubertal children and children with diabetes duration of only 1–2 years; however, they may occur after the onset of puberty or after 5–10 years of diabetes [8]. On the other hand, well-organized and accessible diabetes services can facilitate healthcare and reduce the expenditure due to diabetes complications. For this reason, the organization and quality of the national healthcare system play a relevant role [9]. In 1987 Italy was among the first countries worldwide to introduce legislation to provide diabetes care and implement pediatric diabetes centers in each region with the act “Provisions for the prevention and the cure of diabetes mellitus” [10]. This law predates, by about 5 years, the recognition of special needs of children and adolescents with diabetes by the International Diabetes Federation (IDF). In 2000, the International Society for Pediatric and Adolescent Diabetes (ISPAD) introduced guidelines stating that medical care should cover the whole territory and that all people with diabetes should have access to cost-effective evidence-based care [11]. Since 2001, healthcare and its organization in Italy have been delegated to the 20 individual regions (political and administrative units); the legislative planning and its application vary widely among them [12, 13]. In 2003, the Italian Society of Pediatric Endocrinology and Diabetology (ISPED) developed clinical and organizational guidelines for the management of childhood/adolescent diabetes based on ISPAD guidelines and Italian law [14]. A multidisciplinary, specially trained team was established to assess the performance of each healthcare center. The impact of the new economical crisis in recent years makes this variation more evident and the “spending review” applied to the National Health Care System is matter of concern for many families with a child with diabetes. The aim of this study was to assess the organization of the pediatric care system in Italy, using data obtained from health care professional members of ISPED.

## Materials and methods

In November 2011 a two pages questionnaire was sent by email to 114 ISPED members working in a total of 68 pediatric centers. All the members were invited to send back information regarding the center organization and activities in diabetes care at the date of January the 30th 2012. A center for pediatric diabetes was defined as a place where a child with T1D can be diagnosed and followed by pediatricians with experience in diabetes care, independently of the number of patients followed in that center.

In Italy, since the introduction of law n. 115 (1987), the care of children and adolescents with diabetes is almost exclusively managed by pediatric diabetologists, with a very little contribution by diabetologists for adults. The Annals, published yearly by the national association of adult diabetologists, reported in 2013 more than 500.000 patients, with less than 3 % of subjects under 15 years of age.

The structured questionnaire focused on different issues, including personal information on responder (qualification, degree, specialties, position), department organization, team composition (multidisciplinary or not, full-time or part-time staff working in the diabetes center), activities (number of children in care, maximum age allowed by law, effective maximum age of patients being cared for, number of days dedicated to treating patients/week) and local law (presence of or planned development of laws within the organization’s region).

The Italian National Health System has a departmental organization in order to optimize the use of technical and human resources; each department requires a unique coordination and consists of different units with similar and/or complementary skills working in an integrated way. The complexity of the organizational structure is defined, on the basis of regional laws, by the number and heterogeneity of its resources (human, technological and instrumental), by the importance of the issues addressed, by the relevance of its institutional relations, by the level of its autonomy and of its intersectorality. Complex operational units [UOC] are provided with all technical and professional activities which characterize a specific field, have got managerial autonomy, significant technical and instrumental equipment, and a relevant role in the achievement of the department aims. Simple operational units [UOS] represent functional structures of complex units and derive from specific articulations of clinical activities. Simple departmental operational units [UOSd] are structures created inside the department in order to organize and manage specific activities with responsibility and with professional and organizational autonomy. Lastly, there are ambulatory care services [AC] dedicated to specific health care activities

The recipients were reminded by email if they failed to return the questionnaire in due time. Incomplete questionnaire were gathered and treated by the principal investigator who directly contacted by phone each participant helping him to complete the missing data. Questionnaires coming from members of the same center were merged. When information were not homogeneous, principal investigator directly contacted each participant in order to solve discrepancies. Consistency between the answers was evaluated an it exceeded 90 %. On May 2012 the database was completed. Since UOSd and UOC represented a small number of the sample, they were analyzed together. The distribution of healthcare professionals according to the departmental organization was analyzed using Kruskal-Walis test. The distribution of centers according to patients followed was analyzed using Fisher Exact test. Statistical analyses were performed using R.2.15.3 statistical package. The statistical significance was assessed using a level of probability lower than 0.05.

## Results

A total of 75 (65,7 %) out of 114 questionnaires coming from all the 68 centers were completed. The completeness of this ascertainment was 100 % of all centers following children with diabetes. Overall, 44 centers were classified as an AC, 17 as a UOS, 5 as a UOC, and 2 as a UOSd. The centers distribution according to the region is shown in Table 1; the distribution of health care professionals according to the department organization and the amount of working-time spent on diabetes care is shown in Table 2. The centers classification according to the number of patients is reported in Table 3. The mean number of patients treated was 132 (range 8–600) in centers classified as AC, 397 (range 70–200) in centers classified as UOS and 568 (range 200–1149) in centers classified as UOC-UOSd. Based on this survey, 15,563 patients with T1D, aged less than 18 years, were treated across 68 diabetes centers in Italy. Accordingly, the prevalence of T1D was calculated to be about 1.4 patients per 1,000 people. The characteristics of staffing in these centers, stratified by quartiles of the number of patients, are presented in Table 4. The age limit of patients treated in the participating centers, according to regional law, was reported to be 18 years in 40 centers, 19–22 years in 7 centers, and >23 years in 21 centers. In terms of the actual age of patients treated, it was up to 18 years in 11 centers, up to 19–22 years in 26 centers, and >23 years in 31 centers. The number of weekly days dedicated to treat patients with T1D was 2 for centers classified as AC, 4 for centers classified as UOS, and 5 or 6 for centers classified as UOC/UOSd. Among responders, all declared to be specialized in Pediatrics, 20 % of them both in Pediatrics and Endocrinology. A senior pediatric diabetologist available on call was not present for AC, occasionally for UOS, 24/7 for UOC/UOSd. At the time of this survey, 10 out of 20 regions had introduced supportive legislation for diabetes care (Table 5). In addition, one region (Emilia Romagna) was currently developing appropriate legislation and one region (Trentino) had introduced a memorandum of understanding (Table 5).

**.Table 1:** Distribution of pediatric centers for diabetes in Italy, during the year 2012

Region	OD	UOS	UOSd	UOC	Total number of centers	Total number of patients being treated	Mean number of patients per center
Valle d’Aosta	1				1	35	35
Piemonte	3			1	4	815	204
Lombardia	6	2			8	1965	246
Trentino	1	1			2	365	183
Friuli Venezia Giulia		2			2	253	127
Liguria	3				3	568	189
Veneto	2	1		1	4	768	192
Emilia Romagna	6				6	802	134
Toscana	2	1	1		4	964	241
Marche				1	1	500	500
Umbria		1			1	265	265
Lazio		2		1	3	1619	540
Abruzzo	1	1			2	400	200
Campania		2		1	3	978	326
Puglia	5	1			6	620	103
Basilicata	1				1	20	20
Calabria	10				10	335	34
Sicilia	1	2	1		4	1766	442
Sardegna	2	1			3	2610	870
Total	44	17	2	5	68	15648	230

**Table 2:** Distribution of healthcare professionals in pediatric centers for diabetes according to the department organization and the amount of working-time spent on diabetes care, year 2012

Operators	OD	UOS	UOC/UOSd	*p*
Primary care physicians				
Full time	0 (0–0)	1 (0–1)	2 (2–2.5)	<0.001*
Part time	2 (1–2)	1 (1–2)	1 (0–1)	0.083
Nurses				
Full time	0 (0–0)	0 (0–1)	3 (1.5-3.5)	<0.001**
Part time	2 (1–2)	2 (1–2)	0 (0–1.5)	0.227
Psychologists				
Full time	0 (0–0)	0 (0–0)	1 (0.5-1)	0.002**
Part time	1 (0–1)	1 (1–1)	0 (0–1)	0.466
Dieticians				
Full time	0 (0–0)	0 (0–0)	1 (0.5-1)	<0.001**
Part time	1 (0–1)	1 (1–1)	0 (0–0.5)	0.053

Values are median (1st-3rd quartiles). *p*-values refer to Kruskal-Wallis test and indicate significant differences between groups when <0.05

*Significant differences between groups: OD vs UOS, UOC/UOSd

**Significant differences between groups: OD vs UOC/UOSd

**Table 3:** Distribution of centers according to the number of patients treated

Quartile of the size of the center (patients)	OD number of centers *n* (%)	UOS number of centers *n* (%)	UOC/UOSd number of centers *n* (%)	*p*
1 (<54)	17 (100)	0	0	<0.001
2 (55–115)	13 (76,5)	4 (23,5)	0	
3 (116–309)	10 (62,5)	6 (31,2)	1 (6,2)	
4 (>310)	4 (23,5)	7 (41,2)	6 (35,2)	

Fisher’ exact test

**Table 4:** Staffing of respondents’ pediatric centers for diabetes and its relation to the number of patients treated, year 2012

Quartile of the size of the center (patients)	Mean number of Physicians involved in the care	Mean number of FTE † physicians involved in the care	Centers having a nurse (%)	Mean number FTE† diabetic nurse	Centers having a psychologist (%)	Mean number of FTE † psychologist	Centers having a dietitian (%)	Mean number of FTE † dietician
1 (<56)	1.3	0	88	0,1	64.7	0,1	47.1	0,1
2 (56–115)	1.6	0,2	94	0,4	74.6	0,1	88.2	0.1
3 (116–304)	1.9	0,7	100	0,6	94.1	0,2	94.1	0,2
4 (>305)	2.7	1,5	100	1,6	88.2	0,4	82.4	0,4

Values are an average of the centers in the respective quartile

† FTE indicates appointment in full-time equivalents

**Table 5:** Regional legislation and current application

Region	Regional law available	Regional law applied	Details
Valle d’Aosta	No	n.a.	n.a.
Piemonte	Yes	Yes	Regional law 7 April 2000, no. 34
Lombardia	Yes	Partially	Regional law 30 December 2009, no. 33, ART. 48
Liguria	No	n.a.	n.a.
Veneto	Yes	Partially	Regional law 11 November 2011, no. 24
Trentino	No	n.a.	Protocol agreement
Friuli Venezia Giulia	No	n.a.	n.a.
Emilia Romagna	In progress	Partially	Health and social policies circular 05 September 2003
Toscana	Yes	Yes	Regional law 7 April 2000, no. 34
Marche	Yes	Yes	Regional law 23 February 2009
Umbria	Yes	Partially	Regional law 29 June 2005, no. 1084
Lazio	Yes	Yes	Regional law 4 August 2005, no. 729
Abruzzo	Yes	Yes	Regional law 28 October 2011, no. 920
Molise	No	n.a.	n.a.
Campania	Yes	Yes	Regional law 22 July 2009, no. 9
Puglia	No	n.a.	n.a.
Basilicata	No	n.a	n.a.
Calabria	Yes	Partially	Regional law 18 June 2009, no. 368
Sardegna	No	n.a.	n.a.

## Discussion

This survey described for the first time important features of the 68 centers taking care of children and adolescents with diabetes in Italy during 2012. It also reported an update of the regional laws for youth with diabetes and their application. We can assume that the picture coming to light from this survey gives us complete information about the organization of pediatric diabetes care in Italy, since in our country the care of children and adolescents with diabetes is almost exclusively managed by pediatric diabetologists. For all centers a large variation in geographical distribution, organizational structure and composition of the health care team was observed. Fifty-six percent of centers were formally established with approval from the regional health board [15, 16]. One or two regional centers with few small ambulatory services were identified in 40 % of the regions, while 10 official centers were reported in the Calabria region. No center was identified in the Molise region. These differences could be attributed to the geographical features of the regions. Four different organizational structures were identified (UOC, UOS, UOSd, AC) with 64,7 % of centers classified as AC, 25 % as UOS, 7,3 % as UOC, and 2,9 % as UOSd. As expected, all of the centers following less than 54 patients were organized as AC and with the increasing number of patients followed the organizational complexity of centers increased significantly.

Less than half of the centers had a multidisciplinary team available, as also described by the DAWN Youth report in 2008 [16]. These large geographical and structural differences suggest a huge heterogeneity in the care provided to children and their families. However, despite this heterogeneity, quality of care seems to be of high level if compared with other countries [17].

Since this survey was not designed to investigate the quality of care we are not able to understand if there are differences in clinical outcomes and patient satisfaction between large and small centers.

In terms of legislation, Italian pediatric diabetes centers generally adhere to national/international guidelines and to Italian laws. Several regions support the establishment of diabetes centers based on law no. 115 (1987), 10 out of 20 regions have already introduced appropriate laws and nearly all regional acts support the establishment of specialized pediatric diabetes centers at local level. However, in most cases, the regional legislation is only partially applied.

This is the third study investigating the organization of pediatric diabetes centers in Italy. In the survey performed in 2003 there were 53 active centers, 23 of which (43 %) formally established. In a similar survey performed in 2008, there were 63 active centers, including 25 regional centers and 38 local centers. Considering the current reduction in healthcare expenditure, it is necessary to avoid the breakup of pediatric diabetes centers, which is particularly important considering the rising incidence of the disease. Moreover, each center, especially if classified as UOC and UOSd, should have a minimum number of healthcare professionals who exclusively treat pediatric diabetes patients.

Other countries throughout the European Union are likely to face these challenges, even if they have significantly different pediatric diabetes healthcare systems. Only 19 out of 27 countries have officially recognized centers for pediatric diabetes, while the other countries have centers that provide care to both adults and children, with variable involvement of the different health professionals. The care of children and adolescents with diabetes is mainly provided by pediatricians in Finland, France, Ireland, and the Netherlands, by the general practitioners in Great Britain and Lithuania, by the diabetologists for adults in Portugal, Denmark, and Belgium. In Romania, pediatric and adult diabetologists, as well as pediatricians, are equally involved in pediatric diabetes care [18–24]. The 4th National Survey on diabetes services in the UK highlights several major deficiencies, including inadequate numbers of appropriately trained specialist staff for pediatric diabetes teams, poor psychology services, and insufficient collaboration with adult diabetologists. Although improving in a number of key areas, serious deficiencies are also reported in the 5th National Survey [18–20]. Several problems, including limited clinical support staff relative to international recommendations, are also identified in the first assessment of the pediatric diabetes care performed in the Republic of Ireland in 2008 [21]. Therefore, although there are some differences in the organization and delivery of healthcare for pediatric diabetes, the same challenges are faced by all countries within the European Union. The SWEET (Better Control of Pediatric and Adolescent Diabetes: Working to Create Centers of Reference) project should help to ensure the standards of pediatric diabetes care across Europe, taking into account local factors and promoting internationally recognized guidelines [18, 24]

This study has some limitations that have to be pointed out. First of all the survey is based on data provided by members of the ISPED and not verified by an external auditor. Moreover, information provided by each investigator (e.g., number of patients treated in each center) are obtained from different sources, such as local registry for T1D, clinical database, or uncontrolled archives. In addition, although the survey was conducted in a widespread manner by contacting all members of the ISPED Diabetes Study Group, some members failed to complete the questionnaire for various reasons, including failure to receive the questionnaire or to obtain all of the required information. Finally, during the 7-months study period, it is possible that some organizational changes occurred.

Hopefully, future studies should compare organizational data with clinical information and outcomes (e.g., incidence of severe hypoglycemia, ketoacidosis, death) in order to get useful information for benchmarking and improving the quality of care [21]. In the next survey it will be also useful to collect additional details, including other services provided (e.g., structured therapeutic patient education, use of technology, organization of camps, methods used to assess complications, transition to adult care), and to assess patients’ perceptions of the quality of care through dedicated questionnaires.

The current spending review of the National Health Care System, leading to significant reduction in healthcare expenditure, requires the development of new projects and models of care delivery in order to preserve the quality of care while optimizing costs. The hub and spoke model should probably be considered, particularly where the topography of the area makes transport difficult, and implemented throughout the country in order to provide consistency of care.

## Conclusion

This study shows a large variation in the geographical distribution, organizational structure and composition of the health care team of the Italian pediatric diabetes centers. It also provides new insights and details on the organizational and legislative features of the healthcare system for children and adolescents with diabetes in our country. We believe that the Italian diabetes care organization should be updated taking into account the new economic needs, the increasing incidence of the disease and the impact of the recent immigration waves. Further improvements are also needed in order to assure homogeneity of care throughout the Italian territory.

## Abbreviations

ISPED: Italian Society of Paediatric Endocrinology and Diabetology
T1D: Type 1 diabetes
IDF: International Diabetes Federation
ISPAD: International Society for Pediatric and Adolescent Diabetes
UOC: Complex operational units
UOS: Simple operational units
UOSd: Simple departmental operational units
AC: Ambulatory care services
