# Erratum to: Organization and regional distribution of centers for the management of children and adolescents with diabetes in Italy

**DOI:** 10.1186/s13052-016-0245-8

**Published:** 2016-03-23

**Authors:** Chiara Giorgetti, Lucia Ferrito, Federica Zallocco, Antonio Iannilli, Valentino Cherubini, Study Group for Diabetes of ISPED

**Affiliations:** S.O.D. Pediatric Diabetes, Department of Women’s and Children’s Health, Salesi Hospital, Azienda Ospedaliero-Universitaria, Ospedali Riuniti Ancona, Presidio “G. Salesi”, Via Corridoni 11, 60100 Ancona, Italy

Unfortunately, the original version of this article [1] contained an error. The name of one of the authors from the Study Group for Diabetes of ISPED was included incorrectly and it read “A. Galero” instead of “A. Gaiero” in the Acknowledgements section.

The correct Acknowledgements section has been included in full in this erratum.
